# Unexpected Seizure Activity in the Setting of Lamotrigine Toxicity

**DOI:** 10.7759/cureus.60094

**Published:** 2024-05-11

**Authors:** Danielle Pitter, Samantha Hanley, Hesham Eisa, Yash Nene, Xiangping Zhou

**Affiliations:** 1 Neurology, Upstate Medical University Hospital, Syracuse, USA

**Keywords:** lamotrigine overdose, lamotrigine toxicity, focal epilepsy, seizure medications, seizure activity

## Abstract

Lamotrigine, a widely utilized broad-spectrum anticonvulsant, is commonly prescribed for epilepsy management and bipolar mood disorders. Despite its extensive clinical usage, instances of lamotrigine overdose are underreported. Here, we present a case involving acute encephalopathy and seizure onset following an intentional lamotrigine overdose. This case underscores the importance of recognizing the potential clinical manifestations of lamotrigine toxicity, such as encephalopathy and seizures, emphasizing the necessity for vigilant management of patients receiving this medication.

## Introduction

Lamotrigine, as a phenyltriazine derivative, operates by modulating voltage-gated sodium channels, thereby stabilizing neuronal membranes and inhibiting excitatory neurotransmitter release [1]. Despite its recognized clinical efficacy, misuse or excessive use of lamotrigine may lead to supratherapeutic levels, which can result in significant adverse effects.

Research suggests that instances of lamotrigine overdose are frequently underreported, and when reported, they often exhibit a benign course [2,3]. Lamotrigine poisoning can manifest various central nervous system symptoms, including agitation, dystonia, nystagmus, ataxia, dysarthria, and hypertonia. In severe cases, complications such as seizure occurrence, coma, respiratory depression, intraventricular conduction delays, and even mortality have been documented [3-5]. Here, we present a case involving acute encephalopathy and seizure occurrence in a 28-year-old female patient after an acute overdose of lamotrigine pills.

## Case presentation

A 28-year-old female with a medical history significant for anxiety, major depressive disorder, and a history of localization-related epilepsy, treated with lamotrigine XR 250 mg twice daily, presented to the emergency department (ED) with sudden-onset abdominal pain, nausea, multiple episodes of vomiting, and dizziness following a seizure. She was found lying on the floor with evidence of vomiting by her significant other. The patient admitted to a suicide attempt by ingesting an unknown quantity of her lamotrigine pills.

**Figure 1:**
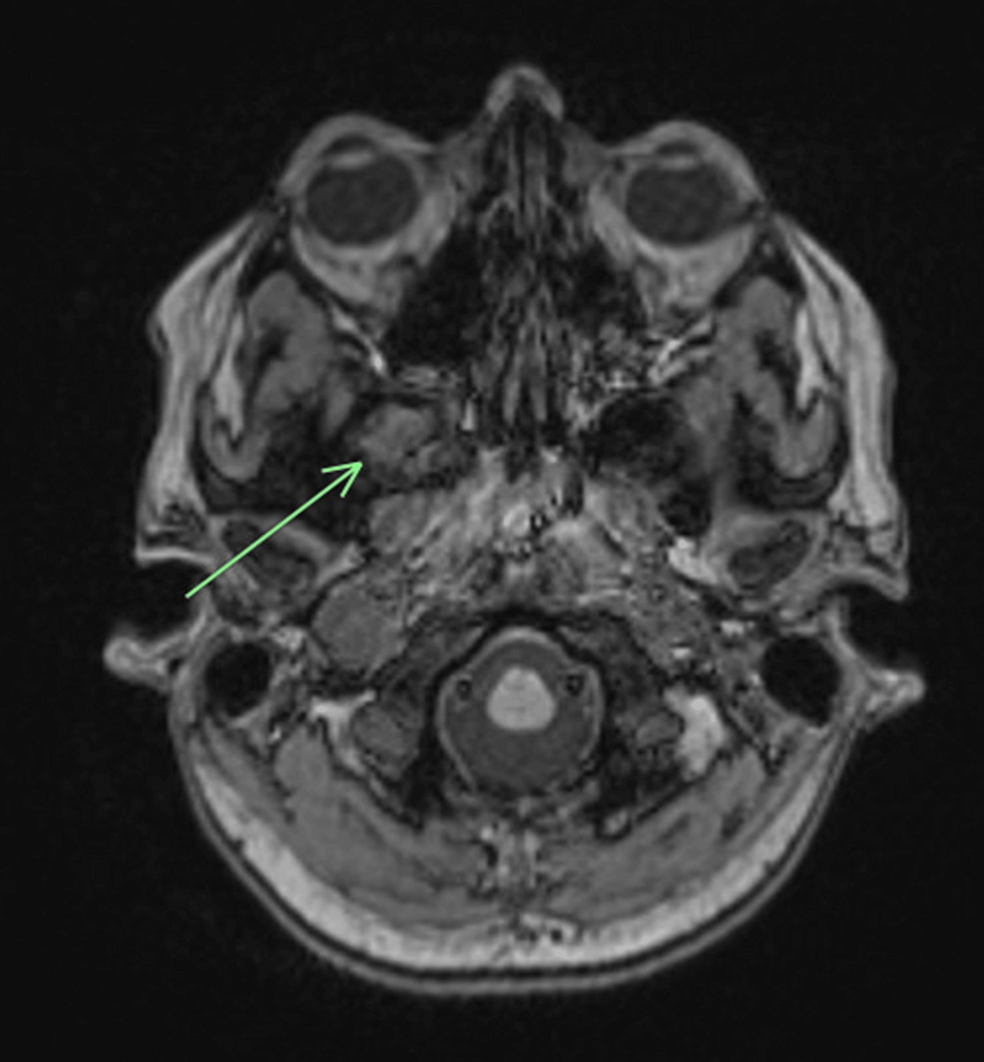
A T1-weighted MRI with contrast shows the known right temporal encephalocele (green arrow).

Upon assessment in the ED, the patient appeared agitated and disoriented with generalized abdominal tenderness on palpation, emesis from her mouth, and bilateral beating nystagmus. Neurological examination revealed direction-changing nystagmus in all directions of gaze, profoundly ataxic upper extremities, brisk reflexes, bilateral ankle clonus, and bilateral Babinski signs. The initial CBC was unremarkable, except for an elevated white blood cell count. A comprehensive metabolic panel and urine toxicology screen were unremarkable. An electrocardiogram (EKG) revealed a prolonged QTc interval of 467 ms, consistent with the previous EKG from six months prior. Serum lactic acid and creatinine kinase (CK) were elevated. Initial CT scans of the abdomen and pelvis with contrast were unremarkable, while CT scans of the head without contrast and MRIs with contrast demonstrated the known right temporal encephalocele without acute pathology (Figure 1). The CT angiography of the head and neck showed no vascular abnormalities. Initial serum lamotrigine levels were elevated (Table 1). An electroencephalogram (EEG) revealed a slow posterior dominant rhythm, with an additional focal slowing in the right posterior temporal region consistent with known temporal encephalocele. Repeat lactic acid and CK levels decreased (Table 1). As the patient's condition began to stabilize, her lamotrigine levels started to decrease within three days (Table 1). She was subsequently discharged to the psychiatric unit and restarted on her home dose of 250 ER mg twice daily, once her levels were within the therapeutic range.

**Table 1:** Initial and repeat lab values upon arrival for lamotrigine toxicity

Labs	Value	Reference
Initial labs		
White blood bell bount	16,400 uL	4,000-10,000 uL
Lactic acid	3.9 mmol/L	0.5-2.2 mmol/L
Creatine kinase	655 U/L	20-180 U/L
Lamotrigine	>40 ug/mL	3-14 ug/mL
Repeat labs		
White blood cell count	17,100 uL	4,000-10,000 uL
Lactic acid	1.4 mmol/L	0.5-2.2 mmol/L
Creatine kinase	1,555 U/L	20-180 U/L
Lamotrigine	29 ug/mL	3-14 ug/mL

## Discussion

Lamotrigine toxicity has been noted in multiple case reports and case series among children and adults. Lamotrigine toxicity can have unintentional causes such as co-ingestion of medications like Divalproex [6] and intentional causes such as this case of a suicide attempt by overdose. Clinical symptoms and severe effects are not commonly noted in patients with this complication [2,7]; however, they do occur. In these patients, vital sign abnormalities such as hypertension, tachycardia, and tachypnea were found [7, 8]. Common clinical symptoms and exam findings would include nausea, vomiting, dizziness, hyperreflexia, myoclonus, flexor plantar response, nystagmus, ataxia, altered mental status, and agitation [7-10]. Rare clinical manifestations of lamotrigine overdose cases include seizures, coma, and respiratory depression [4,8,10], occurring in 0.6%-1.2% of cases [4]. Symptoms such as headaches, dizziness, sweating, and abdominal pain have been found to precede tonic-clonic seizures [11]. Abnormalities in the EKG, such as QRS and QTc prolongation, can also be found in these patients [7,9,11]. 

This case was notable due to the patient's presentation and seizure occurrence despite having elevated levels of lamotrigine on lab work. Dinnerstein et al. [12] presented a very similar case of a 42-year-old woman with a history of localization-related epilepsy, anxiety, and depression who experienced a lamotrigine overdose-induced seizure after a suicide attempt [12]. The woman ingested 4.1 g of lamotrigine and experienced multiple tonic-clonic seizures about one hour post-ingestion. Her initial lamotrigine levels were similar to our case presentation of >40 ug/mL [12]. Griswold et al. [13] presented a case of lamotrigine overdose-induced seizures in a pediatric patient [13]. A three-year-old boy with no medical history presented to the ED after the ingestion of multiple lamotrigine and clonazepam pills. The boy experienced a tonic-clonic seizure, hyperkinesia, and agitation. Lamotrigine levels were 23.2 ug/mL, three hours post-ingestion [13]. Moore et al. [7] presented a case series of 57 patients with lamotrigine toxicity from 2003 to 2012 at an inpatient toxicology center [7]. Nine of the patients, or different age groups ranging from one year old to 57 years old, were found to have lamotrigine toxicity without evidence of other co-ingestants. A third of those patients were found to have seizures with elevated lamotrigine levels [7]. These cases add supportive evidence that seizures are a more common but severe effect of lamotrigine overdoses. 

## Conclusions

This case was clinically significant because the lamotrigine overdose induced a seizure in the patient. The patient's lamotrigine levels were above therapeutic levels, and the patient continued to have seizures. This has been found to be a rare occurrence in multiple case reports and series examining the clinical manifestations of lamotrigine toxicity. Patients having seizures on lamotrigine could be experiencing breakthrough seizures caused by drug ineffectiveness, inaccurate dosing, or patient noncompliance. This case provides support for an additional cause, a lamotrigine overdose. It emphasizes the importance of obtaining and trending patient antiepileptic drug levels when seizures occur, while also stressing the importance of accurate and precise dosing when prescribing antiepileptic drugs.
